# Benchmark of four popular virtual screening programs: construction of the active/decoy dataset remains a major determinant of measured performance

**DOI:** 10.1186/s13321-016-0167-x

**Published:** 2016-10-17

**Authors:** Ludovic Chaput, Juan Martinez-Sanz, Nicolas Saettel, Liliane Mouawad

**Affiliations:** 1 Institut Curie - PSL Research University, Chemistry, Modelling and Imaging for Biology (CMIB), Centre de Recherche, Centre Universitaire, Orsay, Bâtiment 112, 91405 Orsay Cedex, France; 2 Paris-Sud University, Orsay Cedex, France; 3 Inserm, U1196, Orsay Cedex, France; 4 CNRS, UMR 9187, Orsay Cedex, France; 5 School of Pharmacy, University of Caen, Normandy, Boulevard Becquerel, 14032 Caen, France

**Keywords:** Structure-based virtual screening, Docking, Gold, Glide, Surflex, FlexX, DUD-E, Benchmark, BEDROC

## Abstract

**Background:**

In a structure-based virtual screening, the choice of the docking program is essential for the success of a hit identification. Benchmarks are meant to help in guiding this choice, especially when undertaken on a large variety of protein targets. Here, the performance of four popular virtual screening programs, Gold, Glide, Surflex and FlexX, is compared using the Directory of Useful Decoys-Enhanced database (DUD-E), which includes 102 targets with an average of 224 ligands per target and 50 decoys per ligand, generated to avoid biases in the benchmarking. Then, a relationship between these program performances and the properties of the targets or the small molecules was investigated.

**Results:**

The comparison was based on two metrics, with three different parameters each. The BEDROC scores with α = 80.5, indicated that, on the overall database, Glide succeeded (score > 0.5) for 30 targets, Gold for 27, FlexX for 14 and Surflex for 11. The performance did not depend on the hydrophobicity nor the openness of the protein cavities, neither on the families to which the proteins belong. However, despite the care in the construction of the DUD-E database, the small differences that remain between the actives and the decoys likely explain the successes of Gold, Surflex and FlexX. Moreover, the similarity between the actives of a target and its crystal structure ligand seems to be at the basis of the good performance of Glide. When all targets with significant biases are removed from the benchmarking, a subset of 47 targets remains, for which Glide succeeded for only 5 targets, Gold for 4 and FlexX and Surflex for 2.

**Conclusion:**

The performance dramatic drop of all four programs when the biases are removed shows that we should beware of virtual screening benchmarks, because good performances may be due to wrong reasons. Therefore, benchmarking would hardly provide guidelines for virtual screening experiments, despite the tendency that is maintained, i.e., Glide and Gold display better performance than FlexX and Surflex. We recommend to always use several programs and combine their results.

**Graphical Abstract:**
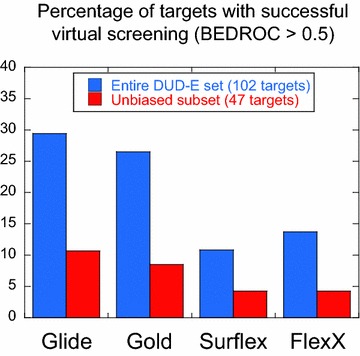
Summary of the results obtained by virtual screening with the four programs, Glide, Gold, Surflex and FlexX, on the 102 targets of the DUD-E database. The percentage of targets with successful results, i.e., with BDEROC(α = 80.5) > 0.5, when the entire database is considered are in Blue, and when targets with biased chemical libraries are removed are in Red.

**Electronic supplementary material:**

The online version of this article (doi:10.1186/s13321-016-0167-x) contains supplementary material, which is available to authorized users.

## Background

The purpose of structure-based virtual screening (VS) is to find compounds with a significant potential to bind a target when a chemical library cannot be fully assessed experimentally. Indeed, biological tests are frequently limited to a small fraction of the chemical library, ranging from tens to few hundreds of compounds. In this respect, VS programs should be able to position active compounds at the top of the ranking, in order to solve what is known as the “early recognition” problem. However, these programs present variable performances and their comparison was described to be useful “to guide the choice of the most performing tools and protocols” in drug design [1].

Since the beginning of the docking and VS era, benchmarks were done to compare the performance of the programs, either by evaluating their ability to reproduce the pose of the crystal structure of known ligands [2, 3] or by studying their efficiency to discriminate between active and inactive compounds [4–6]. To achieve the latter benchmarks, a database with several active and inactive compounds is required. The DUD database [7] (Directory of Useful Decoys) had been specifically designed for this purpose in 2006. It included 40 targets (of which 30 enzymes) and a total of about 3000 active compounds and 36 fold more decoys. This database was used for several benchmarks of VS programs [8–13], either to compare the programs performance or to improve the scoring functions and the docking procedures. However, despite the seriousness of its construction, DUD was criticized because of some biases, namely the difference of the mean formal charge between actives and decoys [14], the similarity between the actives [15] and the small number of different targets [16]. This criticism brought Mysinger and al. [17] to release in 2012 a new version of the DUD database, DUD-E, “E” standing for “enhanced”. In DUD-E, these problems were addressed first by increasing the number of proteins from 40 to 102 and diversifying them (only 61 proteins were grouped in 6 families: GPCR, Kinases, Nuclear Receptors, Proteases, Cytochrome P450 and Ion Channels). Second, the average number of ligands per target was increased from 98 in the old version to 224 in the new one and their diversity was based on their Bemis–Murcko atomic framework clustering. Finally, the number of decoys was increased from about 36 in DUD to 50 decoys per ligand in DUD-E, corresponding to about 2 % actives for each target, and their properties improved to have the same average net charge as the actives. With the total number of compounds exceeding 1.4 million (22,886 actives and 1,411,214 decoys), DUD-E is one of the largest databases publicly available that offers the possibility to assess VS programs efficiency in discriminating ligands from inactive compounds.

The benchmarks of programs were criticized [15] because their results depend on the calculation setup, the metrics used to evaluate the performance, the choice of the targets and the active/decoys selections. In this article, we use the DUD-E database to perform a benchmark of four of the most popular docking programs [18], Gold [19], Glide [20], Surflex [21] and FlexX [22]. Our key objective is not only to compare their performance but mainly to determine what would influence it for each of them. The question addressed here is about the relationship between the performance of these programs and the protein target properties and/or the small molecules properties. For this purpose, we started by evaluating the programs performance using two metrics in three specific cases, then monitored the correlation between this performance and the target or the chemical library properties.

## Results and discussion

The virtual screening calculations were carried out using the four programs cited above, Gold, Glide, Surflex and FlexX, applied to the 102 targets of the DUD-E database, with their 102 chemical libraries. To evaluate the quality of the VS results, the Docking Enrichment and BEDROC metrics, which are two of the most widespread measures, were used.

### Overview of the programs performance

#### Docking enrichment

The docking enrichment (DE) is an intuitive parameter, easy to apprehend. It represents the percentage of true positives found (*h*) among all the active molecules (*n*) for a given percentile of the top-ranked compounds (*x* *%*) of a chemical library:

1 $$ DE_{x\% } = \frac{h}{n} $$

**Fig. 1:**
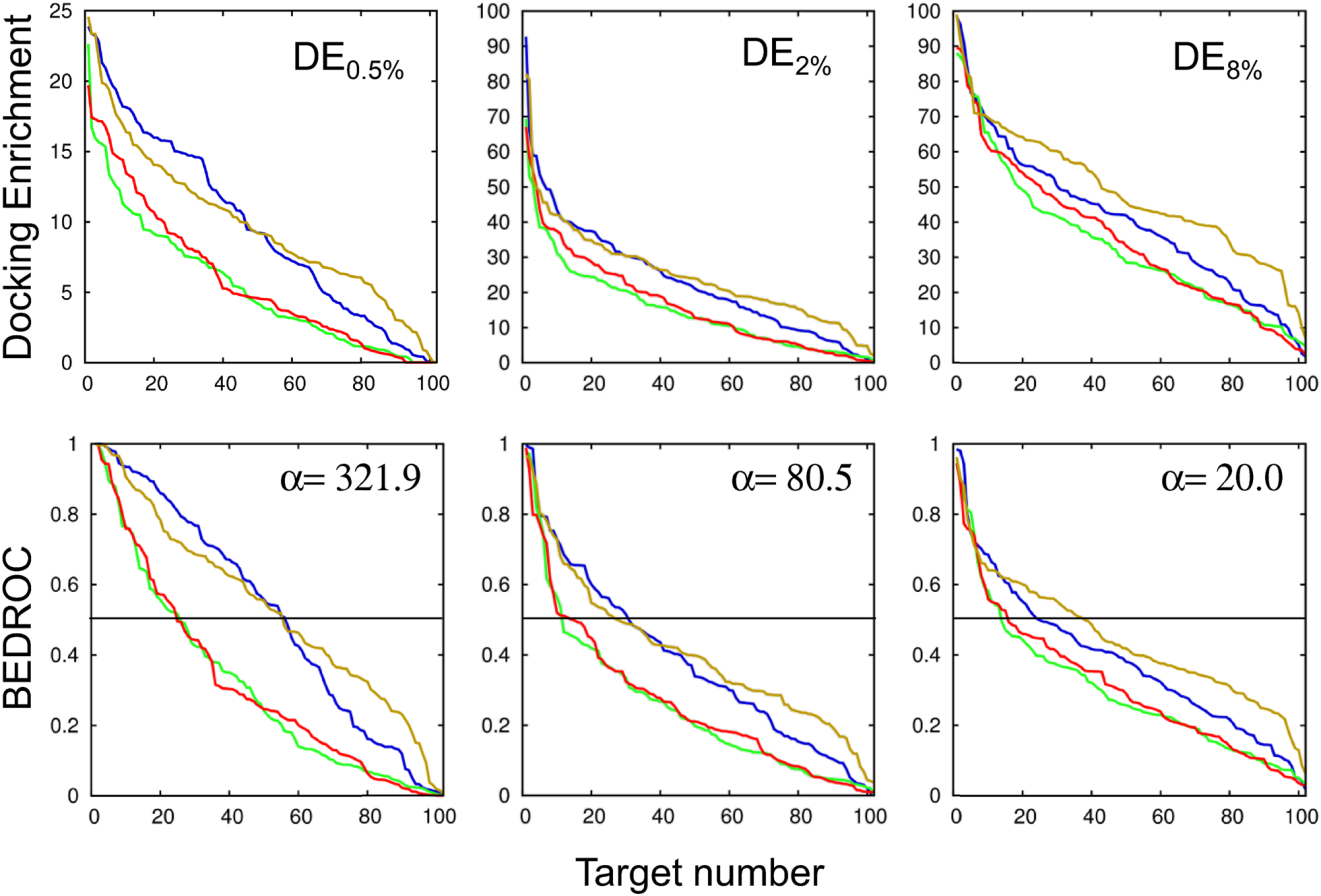
Two metrics to evaluate the performance of the programs with respect to the target number. *Upper panels*: the docking enrichments (DE) for three different percentiles of the chemical libraries, 0.5, 2 and 8 %. *Lower panels*: BEDROC scores for three α values, 321.9, 80.5 and 20.0. For each curve, the results are sorted in the descending order of DE or BEDROC, which means that the target number does not point to the same protein. The curves of Gold are in *yellow*, Glide in *blue*, Surflex in *green* and FlexX in *red*. In the *lower panels*, the *horizontal lines* represent BEDROC score = 0.5

DEs for the 0.5, 2, and 8 % top-ranked compounds were calculated (Fig. 1, upper panels). We chose to focus on these percentiles because 2 % represents the proportion of active compounds in the DUD-E database, and 0.5 and 8 % correspond to 4 times smaller and higher proportions, respectively. The value of 0.5 % allows us to evaluate the performance of the programs in the early recognition VS, which is important when only few compounds can be tested experimentally, whereas 8 % is interesting for the cases where relatively high-throughput experiments are available. Since the active/decoy ratio is about 2 % in all libraries, the best DE achievable for the 2 % percentile, and a fortiori for the 8 %, is 100 %. It drops to 25 % when the chosen percentile is 0.5 %.

From these results, we observe that for the 50 % targets with the best DE, there is a gap between Glide and Gold on the one hand and Suflex and FlexX on the other hand. This gap is clear for *x* *%* = 0.5 %. It reduces for *x* *%* = 2 % and then a new gap appears for *x* *%* = 8 %, between Gold and the three other programs.

#### BEDROC score

The BEDROC score [23] is more complex and less intuitive than the DE, nevertheless, for a given protein, it is not limited to a percentile of the chemical library but it takes into account all the compounds. However, this score can be modulated by the weight given to the top-ranked compounds, by using a parameter α.

2 $$ BEDROC = \frac{{\sum\nolimits_{i = 1}^{n} {e^{{ - \alpha r_{i} /N}} } }}{{R_{a} \left( {\frac{{1 - e^{ - \alpha } }}{{e^{\alpha /N} - 1}}} \right)}} \times \frac{{R_{a} \sinh \left( {\alpha /2} \right)}}{{\cosh \left( {\alpha /2} \right) - \cosh \left( {\alpha /2 - \alpha R_{a} } \right)}} + \frac{1}{{1 - e^{{\alpha \left( {1 - R_{a} } \right)}} }} $$

where *n* is the number of actives, *N* the total number of compounds, *R*_*a*_, the ratio of actives in the chemical library (*R*_*a*_ = *n/N*) and *r*_*i*_, the rank of the *i*th active given by the program. For α = 80.5, the 2 % top-ranked molecules account for 80 % of the BEDROC score. The α value can be modulated so that 0.5 % (α = 321.9) or 8 % (α = 20.0) of the top-ranked molecules account for 80 % of the score. The BEDROC scores using the three α values were calculated for the 102 protein targets based on the VS results obtained from the four programs. The curves are represented in Fig. 1, lower panels, and the values for α = 80.5 are reported in Additional file 1: Table S1. It can be observed that, as expected, the BEDROC curves compare qualitatively well with the corresponding DE curves, i.e., BEDROC(α = 321.9) corresponds to DE_0.5 %_, BEDROC(α = 80.5) to DE_2 %_ and BEDROC(α = 20.0) to DE_8 %_. Therefore, the assessment of the docking programs based on this metric is similar to DE, showing the pertinence of the choice of the α values.

The comparison of the BEDROC scores of the two best-performing programs, Gold and Glide, shows that their relative performance depends on the value of α. For α = 321.9, Glide has higher scores than Gold for about 50 % of the best-ranked protein targets. For α = 80.5, this percentage drops to about 30 % and for α = 20.0, it drops to <10 %. This would suggest that Glide is a better candidate for early recognition problems.

Focusing on the proteins with the best BEDROC scores, greater than 0.5 (see Fig. 1, lower panels), and comparing their numbers instead of their scores, show that the number of proteins with the highest scores is similar for Glide and Gold, when α = 321.9 and α = 80.5, and significantly higher for Gold, when α = 20.0. In all cases, the number of these proteins is lower with Surflex and FlexX. This is summarized in Additional file 1: Figure S1.

These results are based on the distribution of the BEDROC scores and their corresponding number of proteins, but in this comparison, the scores of different programs do not necessarily refer to the same target. The analysis of the BEDROC scores of each target shows that, although the overall scores obtained with Surflex or FlexX are lower than those with Glide and Gold, for a particular target the former two programs may outclass the latter ones. In order to compare the performance of the programs based on their BEDROC scores for the same target, we adopted a metric similar to what was done in our previous study [24]: the net balance between the number of protein targets for which program(a) has higher BEDROC scores than program(b) $\left( {n_{p}^{{{\text{BEDROC(a) }} > {\text{ BEDROC(b)}}}} } \right)$ and the number of targets for which program(a) has lower BEDROC scores than program(b) $\left( {n_{p}^{{{\text{BEDROC(a) }} < {\text{ BEDROC(b)}}}} } \right)$. This net balance is reduced to a percentage, Δ*p*(a/b), by taking into account the total number of targets, *N*_*p*_.

3 $$ \Delta p ( {\text{a/b)}} = \frac{{n_{p}^{{{\text{BEDROC(a) }} > {\text{ BEDROC(b)}}}} \, {-} \, n_{p}^{{{\text{BEDROC(a) }} < {\text{ BEDROC(b)}}}} }}{{N_{p} }} $$

**Fig. 2:**
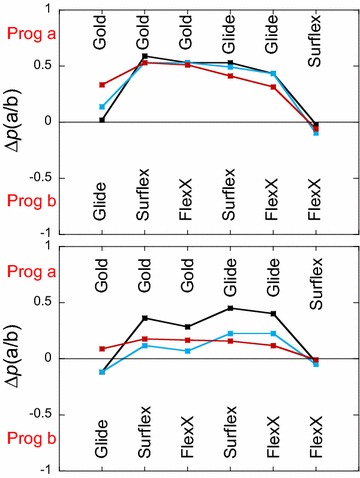
Pair-wise comparisons of the programs performance. The net balance Δ*p*(a/b) represents the fraction of proteins for which program **a** obtained a better BEDROC score than program **b** minus the fraction of proteins for which it is program **b** that obtained the better score. Program **a** is represented in the upper half of* each panel* and program **b** in the lower half, i.e., Δ*p*(a/b) > 0 if the net balance is in favor of program **a** and negative otherwise. *Upper panel*: Δ*p*(a/b) when all proteins are considered. *Lower panel*: Δ*p*(a/b) when only proteins with BEDROC scores >0.5 are considered. *Black curves*, for α = 321.9, *blue*, for α = 80.5 and *red*, for α = 20.0

The interest of the net balance is that it is based on the comparison of the BEDROC scores for each target and that the plots are easy to interpret: when Δ*p*(a/b) > 0 program(a) has a better net performance than program(b) and when Δ*p*(a/b) < 0 it is the other way round. In Fig. 2 (upper panel), are reported the Δ*p*(a/b) values based on all the BEDROC scores, showing that Gold > Glide ≫ FlexX ≥  Surflex for all the three α values considered in this study. Notice that the important gap between Gold and Glide on the one hand and Surflex and FlexX on the other hand is confirmed here, since the net balance is in favor of the two former programs for about 50 % of the protein targets. In Fig. 2 (lower panel), Δ*p*(a/b) is calculated considering only the targets that obtained BEDROC scores greater than 0.5 by at least one of the two compared programs (a/b). In this case, Glide obtained better BEDROC scores than Gold for about 12 % excess targets when α = 321.9 and α = 80.5. However, when α = 20.0, Gold obtained better BEDROC scores than Glide for about 8 % excess targets. Considering FlexX and Surflex, their gap with Gold and Glide is diminished and the difference between them is still small and in favor of FlexX. Therefore, for rather early recognition VS (α = 321.9 and α = 80.5), when the programs that succeed (BEDROC score > 0.5) are only considered, their performance may be classified as follows, Glide > Gold > FlexX ≥ Surflex.

The following sections will focus on what determines the performance of these programs and what would be the relationship between this performance and exogenous elements, such as the family of proteins to which the target belongs, the composition of the binding site or some descriptors of the chemical libraries.

### Relationship between the programs performance and the protein target characteristics

#### Subsets of protein families

We started by determining the protein families of the DUD-E database. These families were only partially based on the target subsets established originally in DUD-E [17], because we noticed that, in this database, the proposed families might contain targets with too low structural homologies. Therefore, keeping the DUD-E classification in mind, we determined the protein families according to three criteria: the sequence identity, the protein function and the protein fold. The sequences of all 102 proteins of the DUD-E were aligned using the Clustal Omega method [25]. All pairs of proteins with more than 20 % sequence identity were analyzed. If these pairs shared, in addition, the same protein function and a similar protein fold, they were grouped in the same family. Nine families of various sizes were obtained, made of 2–26 members, and gathering a total of 58 proteins over the 102 targets of DUD-E. The composition of these families is presented in Additional file 1: Table S2.

#### Performance of the programs with respect to the protein families

**Fig. 3:**
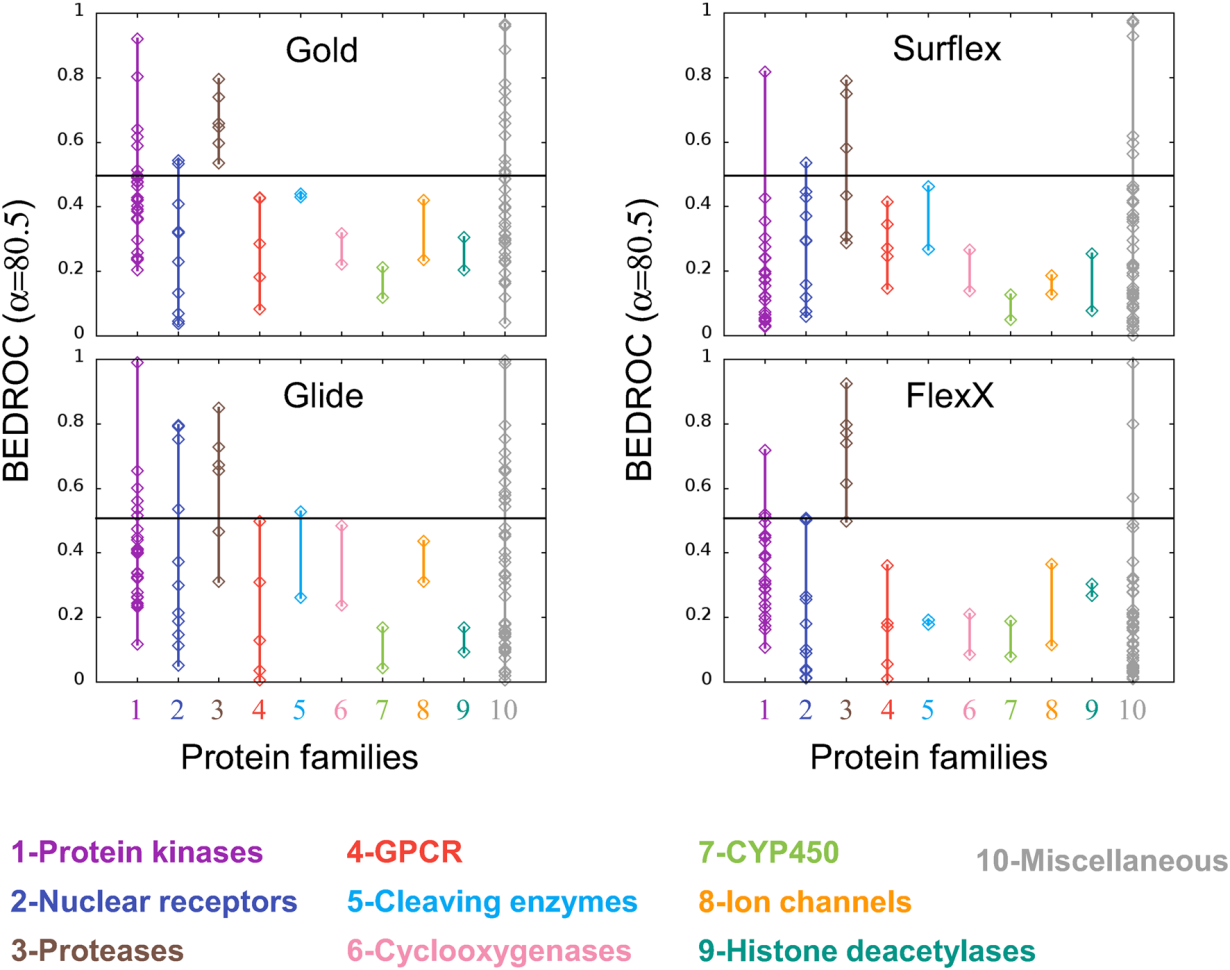
Programs performance with respect to the protein families. For each program, the BEDROC scores (α = 80.5) of all targets are ordered by protein families. The *color code* is given below the plots. As in Fig. 1, the *horizontal lines* represent BEDROC score = 0.5

For each program, the BEDROC scores with α = 80.5 are reported with respect to the target families in Fig. 3. The scores obtained with α = 321.9 and α = 20.0 are given in Additional file 1: Figure S2. In Fig. 3 some similarities are observed between the results of the four programs. They all failed in obtaining high BEDROC scores for some families, such as the G protein-coupled receptors (GPCR), the cleaving enzymes, the cyclooxygenases, the cytochromes P450 (CYP450), the ion channels and the histone deacetylases. For the nuclear receptors, few members of the family obtained scores over 0.5 (4/11 with Glide, 2/11 with Gold and FlexX and 1/11 with Surflex), similarly to the protein kinases (with scores over 0.5 for 6/26 proteins with Gold and Glide, 4/26 with FlexX and 1/26 with Surflex). In the latter family the best score was obtained by the same protein, wee1, with all programs. For the family of proteases, all four programs yielded good results, with BEDROC scores over 0.5 (6/6 with Gold, 5/6 with FlexX, 4/6 with Glide and 3/6 with Surflex).

Based on these results, it is difficult to assert that there is a correlation between the program performance and the protein families. However, for the protease family proteins, Gold and FlexX are the best performing programs, and for protein kinases, Gold and Glide give an average result.

Considering the good performance of Gold for the protease family, it could be possible that this family was used as a training set for Goldscore, but this is not the case. Indeed, none of the proteins of this family was a member of the training set published by Jones et al. [26].

#### The protein binding site properties

**Fig. 4:**
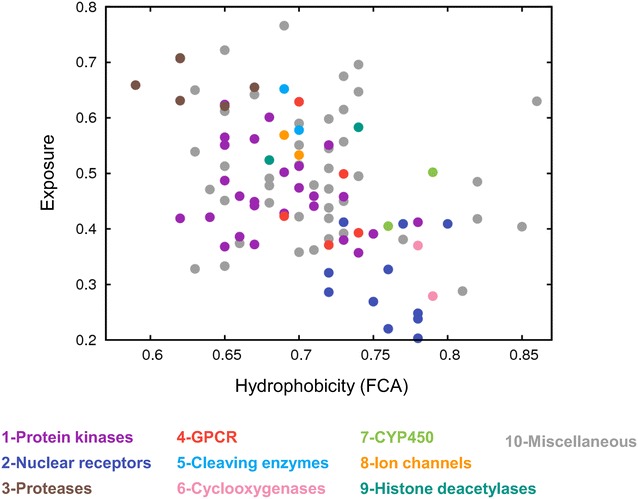
Characteristics of the protein cavities. For each target, the exposure of the cavity is represented *vs* its hydrophobicity and colored according to the target family. The *color code*, which is given below the plot, is the same as in Fig. 3

To find out which properties of the protease family gave rise to such good scores, especially with Gold, the characteristics of the binding site for all the protein targets of the DUD-E database were investigated. Two properties would characterize well a protein binding site, its exposure to the solvent and its hydrophobicity. The exposure property was calculated using the SiteMap algorithm [27] from the Schrödinger suite, where it is described to “provide a measure of how open the site is to solvent”. Regarding the hydrophobicity of the binding site, it did not seem to us appropriate to consider the nature of the residues (hydrophobic/hydrophilic), because even a hydrophilic residue may establish hydrophobic contacts with a small molecule and vice versa. In addition, for some targets, co-factors and metals are inherent parts of the site. Therefore, we preferred to consider the fraction of carbon atoms (FCA) that are located in the binding site at less than 4 Å from the protein surface (see “Methods” section for details), because this is more representative of the number of hydrophobic contacts that may be established with the ligand. These binding site characteristics are plotted in Fig. 4, where for each target, its exposure versus its FCA is reported, with the corresponding dot colored according to the protein family to which the target belongs. In this Figure, we observe first that, as expected, there is a significant correlation between the exposure and the fraction of carbon atoms of the binding site, with a coefficient of −0.4 and a *p*-value lower than 10^−4^. This comforts our definition of the degree of hydrophobicity of the binding site, since the more hydrophilic is the site, the more it is open and exposed to solvent. Second, the protein families are relatively well grouped in the plot, showing similarities between their binding sites, although the families were defined based on the sequence alignment and the 3D structure of the protein in its entirety. Finally, the proteases, which obtained generally good BEDROC scores, are all located in the upper left corner of the figure corresponding to hydrophilic binding sites well opened and exposed to solvent, whereas, on the opposite side of the plot, corresponding to hydrophobic and closed binding sites, are the nuclear receptors, which obtained rather low BEDROC scores, except with Glide. Therefore, this result seems to indicate that there is some correlation between the two properties of the binding sites on the one hand and the performance of the programs on the other hand. To verify this assumption in the case of α = 80.5, we used similar plots of the binding site characteristics (i.e., exposure *vs* FCA), where we colored in red all the targets corresponding to BEDROC scores over 0.5, not only those belonging to the identified families (Additional file 1: Figure S3). For the four programs, the red dots are distributed all over the plots, showing that for good BEDROC scores, there is no evidence of significant correlations between the program performance and these characteristics of the protein binding site. However, when all BEDROCs are considered, not only those over 0.5, small correlations are observed between the performance of Gold and FlexX on the one hand and the hydrophobicity of the binding site on the other hand, the correlation coefficients being −0.29 and −0.32, respectively (with *p*-values <10^−2^). This correlation was also observed for FlexX in other studies based on seven targets [28, 29]. For Glide and Surflex, there are no such correlations (with *p*-values equal 0.05 and 0.03, respectively). Considering the exposure of the binding site, there is no correlation between this property and any of the program performances. In all that follows, correlations are considered as significant when their *p*-value ≤10^−4^, as questionable when 10^−4^ < *p*-value ≤10^−2^ and inexistent when *p*-value >10^−2^, because we noticed that, in our case, the correlations were visually observable in the plots only for *p*-values ≤10^−4^.

For α = 321.9, the small correlation between the hydrophobicity of the binding site and the performance of FlexX is maintained (correlation coefficient of −0.32 and *p*-value <10^−2^) but not that of Gold, whereas for α = 20.0, both program correlations, although questionable, are comforted with coefficients equal to −0.36 for Gold and −0.32 for FlexX and *p*-values <10^−3^.

In addition, we considered the size of the binding site. This size, as calculated by SiteMap, is well-correlated to the exposure of the site (*r* = −0.4 and *p*-values <10^−4^). Therefore, it does not constitute an independent variable. We instead took into account the total number of atoms in the binding site that are at less than 4 Å from the protein surface. The interest of this property is that it represents the total number of possible contacts that the protein may establish with the ligand that binds to its site. The performance of the four programs (their BEDROC scores) did not correlate with this variable (all the *p*-values >10^−2^), showing that the putative number of contacts does not determine the performance of any of the four programs studied here.

#### The quality of the protein structure

**Fig. 5:**
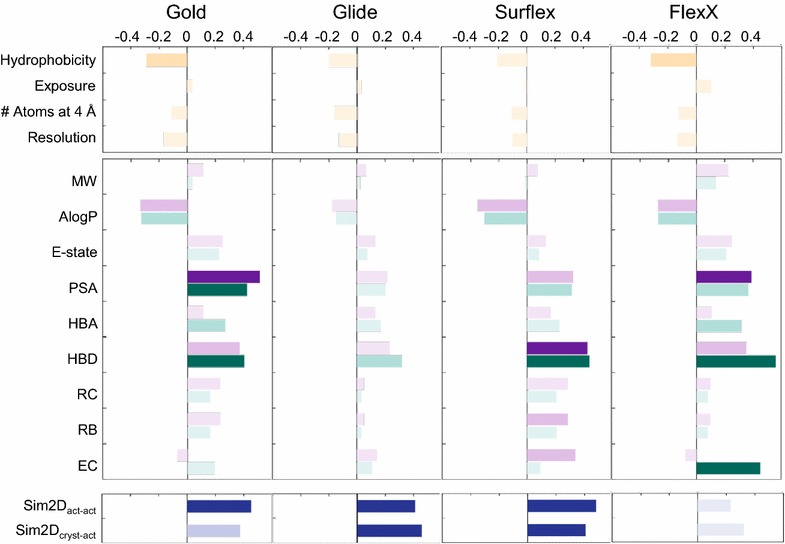
Correlations between the BEDROC scores (α = 80.5) and the properties of the targets and the small molecules. *Upper panels*: correlations with the protein properties, in orange, i.e., with the hydrophobicity of the cavity, its exposure and its number of atoms at 4 Å from the surface, in addition to the crystal structure resolution. *Middle panels*: correlations with the small molecule properties, in* purple* for the actives and* green* for the decoys, i.e., the molecular weight (MW), the octanol/water partition coefficient (AlogP), the electrotopological state (E-state), the polar surface area (PSA), the number of hydrogen bond acceptors (HBA), the number of hydrogen bond donors (HBD), the ring count (RC), the number of rotatable bonds (RB) and the embranchment count (EC). *Lower panels*: correlations of the BEDROC scores with the 2D fingerprints similarities between the actives (Sim2D_act-act_) and between the actives and the crystal structure ligand (Sim2D_cryst-act_), in* blue*. In* all panels*, the* darkness* of the* colors* depends on the *p*-value of the correlation, dark when *p*-value ≤10^−4^, medium light when 10^−4^<*p*-value ≤10^−2^ and light when *p*-value >10^−2^

Finally, we investigated the possibility that the performance of the VS programs may depend on the quality of the protein structure. For this purpose we calculated the correlation between the program BEDROC scores and the resolution of the protein structure taken from the PDB file. No significant correlation was observed. All the results concerning the correlations between the BEDROC scores for α = 80.5 and the protein characteristics are summarized in Fig. 5, upper panel. For α = 321.9 and α = 20.0, see Additional file 1: Figure S4.

### Relationship between the programs performance and the small molecules characteristics

#### The small molecules properties

**Fig. 6:**
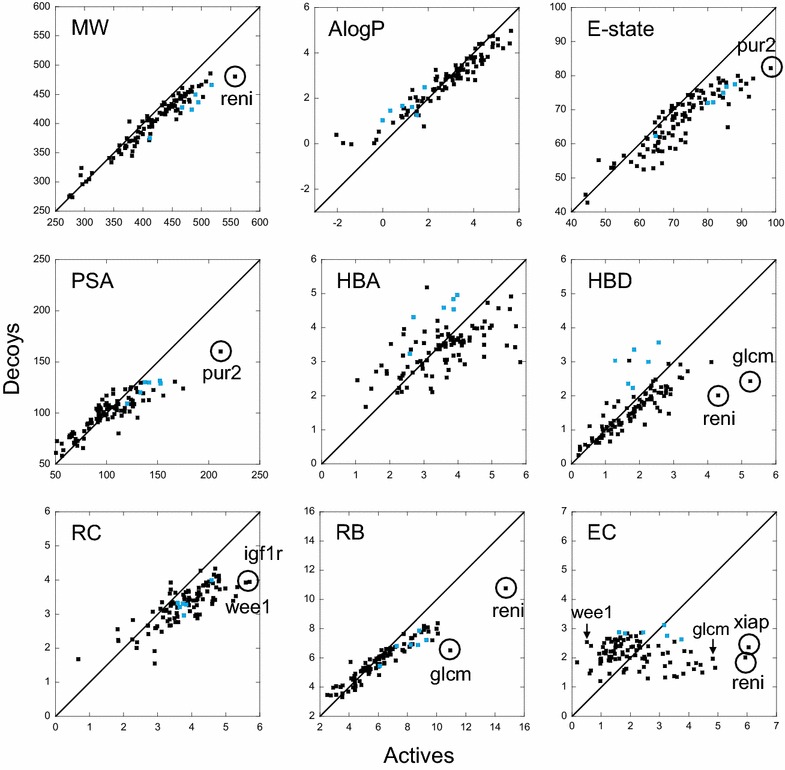
Properties of the small molecules.* Each plot* corresponds to one of the nine properties considered in this study. In* all panels*, for each target, the average property of the decoys is plotted versus the average of the same property for the actives. The *blue dots* correspond to the six proteins of the protease family. The *diagonal line* represents the ideal situation where the average property for the actives and decoys are equal. For some properties, the most stand-out targets are* encircled*

Since no clear correlation was observed between the protein binding site and the performance of the programs, one may ask if such correlations exist between the performance and the chemical libraries properties. To monitor this aspect we calculated 365 physicochemical properties for each small molecule using the Canvas program [30] from the Schrödinger suite. Based on these calculations, for each of the 102 chemical libraries, corresponding to the 102 targets of the DUD-E database, we considered separately two molecule subsets, the true actives and the decoys, and computed the averages of the properties for each of these subsets. Only the most relevant and non-redundant (despite some correlations between them) of these average properties were kept for the analysis: the molecular weight (MW), the octanol/water partition coefficient (AlogP), the electrotopological state (E-state), the polar surface area (PSA), the number of hydrogen bond acceptors (HBA), the number of hydrogen bond donors (HBD), the ring count (RC), the number of rotatable bonds (RB) and the embranchment count (EC). The electrotopological state describes the intrinsic electronic state of the atom, taking into account the electronic influence of all other atoms in the molecule [31]. The embranchment count was designed to account for the number of all atoms that establish three or more bonds, thus creating ramifications (see “Methods” section). Note that some of the nine properties considered here were taken into account by the authors of the DUD-E database for the construction of the decoys (MW, miLogP, RB, HBA and HBD) [17]. For the 102 targets, the average properties of true actives and decoys were compared (Fig. 6). A more or less important systematic drift was observed: for the high values of the property, the actives reach generally higher average values than the decoys. We will see below that this bias may play an important role in the good performance of some programs.

#### Correlation between the small molecules properties and the programs performance

The correlations between the program BEDROC scores and the nine properties of the true actives or the decoys were calculated. The results are summarized in Fig. 5, *middle panel* for α = 80.5, and Additional file 1: Figure S4 for α = 321.9, and α = 20.0. They are qualitatively similar for the three α values, therefore the analysis is focused on the results of α = 80.5. Glide performance is not correlated with any of those properties, whereas the three other programs performances, Gold, Surflex and FlexX, are significantly correlated to two or three properties among the following: PSA, HBD and EC. When the BEDROC scores of a program are correlated with PSA or HBD of the actives, they are also more or less correlated to the same property of decoys, which is not the case for EC. This is due to the fact that, for PSA and HBD, the average values of the active compounds are highly correlated to those of the decoys, with *r* = 0.89 for the former and *r* = 0.75 for the latter, whereas for EC there is no correlation between the actives and the decoys (*r* = −0.15).

Let us consider separately each of the three properties, PSA, HBD and EC, and analyze the reasons for their correlations with the programs performance for α = 80.5.

Gold performance is correlated to both PSA_actives_ (*r* = 0.51) and PSA_decoys_ (*r* = 0.42), and in addition, to the difference between these two properties, ∆(PSA) (*r* = 0.46, *p*-value <10^−4^). Therefore, the drift in the PSA property observed in Fig. 6, and more particularly the PSA of decoys, seems to have a significant influence on the performance of Gold. For FlexX, the correlation with PSA is mainly with PSA_actives_ (*r* = 0.39, *p*-value <10^−4^), the correlation with ∆(PSA) being small (*r* = 0.29, *p*-value = 3 × 10^−3^). This indicates that the program is sensitive to polarity, since it performs better for polar ligands in polar cavities (the correlation with the cavity hydrophobicity is −0.32). The protease family had the most hydrophilic cavity (see Fig. 4) and obtained good BEDROC scores from FlexX, therefore we considered the possibility that the FlexX-PSA_actives_ correlation may be due in part to this family. To verify it, we ruled out the protease family and calculated the correlation again. The result showed a clear decrease of the FlexX-PSA_actives_ correlation (*r* = 0.28, *p*-value = 3×10^−3^), which got out of the range of what we retained as significant correlations, i.e., *p*-value ≤10^−4^. None of the other families had a similar effect and their absence did not diminish the correlation. It may even be the contrary, as for the example of the protein kinase family, whose absence increased the correlation to 0.46. Although it may seem that the actives play an important role in the performance of FlexX, a property of the decoys is still more decisive, as can be seen in the next section. The highest correlation of FlexX (*r* = 0.56, *p*-value ≤10^−4^) is with HBD_decoys_.

The case of HBD is of the most interesting. This property is correlated to Gold, Surflex and FlexX performances mainly through the decoys. Due to the covariance term of a correlation, the most important contribution to this correlation is due to the values that are the farthest from the average. For the decoys, this is the case of the protease family, as observed in Fig. 6. To verify the importance of this family, we ruled it out and calculated the correlations again. The results showed that all correlations with HBD_decoys_ were significantly diminished, which means that increasing the number of HBDs for the decoys of the protease family improves the performance of Gold, FlexX and Surflex. But the protease binding sites are between 30 and 50 % richer in hydrogen bond acceptors than in hydrogen bond donors. Therefore, the fact that the decoys are richer in HBDs than the actives should normally favor the binding of the decoys, increasing the number of false positives. This should contribute to luring the docking programs instead of improving their performance. So, how can the good performance of these programs be explained in the case of the proteases? To answer this question, the observation of Fig. 6 shows that, for the protease family, similarly to HBDs, HBA_decoys_ are higher than HBA_actives_. Knowing that, for this family, there are for the decoys on average 4.4 HBA/molecule and only 2.9 HBD/molecule, the repulsive interactions between the decoys and the protease cavities are greater than the attractive ones, which facilitates the discrimination between the actives and the decoys. Thus, for the protease family, the increase of HBA_decoys_, which accompanies the increase of HBD_decoys_, is likely responsible for their good BEDROC scores. Therefore, despite what correlations suggest, it is not directly the increase of HBD_decoys_ that is responsible for the performance of the three programs but this good performance may be a consequence of the effect of HBA_decoys_ of the protease family.

Concerning the embranchment count, FlexX performance is highly correlated with EC_decoys_. By ruling out the families, one at a time, we observed that this correlation is mainly due, again, to the proteases. There is no rationale to explain the contribution of this family to the correlation, since the cavity in these proteins is rather open and exposed to solvent and therefore, the high number of embranchments in the decoys is not expected to help discriminating them. However, since the FlexX BEDROC scores of these targets are high, due to their important number of HBA_decoys_, the contribution of the proteases to the FlexX-EC_decoys_ correlation may be fortuitous.

From all these correlations we may conclude that the good performance of Gold, FlexX and Surflex is mainly due to the construction of the decoys. This is particularly true in the case of the protease family, where the decoys are rich in HBA while the cavities are rich in HBA as well.

#### Programs performances for the stand-out targets: the chemical library biases are not sufficient…

Since the programs good performances seem to be mainly due to decoys, it would be interesting to analyze the BEDROC scores obtained by the targets that stand out from the others, i.e. the targets for which the differences between the average properties of the actives and the decoys are the largest. From the preceding section, we may expect that such targets would obtain good BEDROC scores. Observation of Fig. 6 shows that the number of these stand-out targets is six: two protein kinases, wee1 and igf1r (which stand out in the ring counts, RC), one cleaving enzyme, reni (in MW, HBD, RB and EC) and three miscellaneous proteins, pur2 (in PSA and E-state), glcm (in HBD and RB) and xiap (in EC). Interestingly, although Glide performance is not correlated to any of these properties, all six stand-out targets obtained rather good BEDROC scores with this program (see Additional file 1: Tables S1 and S3). Note that pur2 is the target that obtained the highest BEDROC scores (between 0.97 and 1.0) with all programs. Its cavity is hydrophilic (here we consider the complementary of hydrophobicity, i.e., 1-FCA = 0.345) but not much exposed to the solvent (Exposure = 0.374) and its chemical library is such that the average polar surface area (PSA) of the active compounds is 32 % bigger than that of the decoys, favoring the good performance of all four programs. It is interesting to compare the case of pur2 with that of another target, mk01, which is not a stand-out protein, all its decoys properties being close to those of the actives, but which shares with pur2 the same cavity characteristics (1-FCA = 0.353 and Exposure = 0.368). Contrary to pur2, mk01 obtained poor BEDROC scores from all programs, ranging between 0.03 and 0.46, which tend to demonstrate the importance of the decoys construction compared to the cavity characteristics.

The protein kinases, wee1 and igf1r, are the stand-out targets for the RC criterion. Wee1 is the target that obtained the highest BEDROC scores among all protein kinases, with the four programs (between 0.72 and 0.99), whereas igf1r obtained only medium scores (between 0.49 and 0.52) except with Surflex, which was poor (0.20). Both proteins have similar cavities, rather flat, which better accommodate planar compounds, and thus the difference between the ring counts (RC) of actives and decoys (33 % for wee1 and 35 % for igf1r) favors the good discrimination between them. Therefore, to explain the difference of performance of the programs for these two targets, we can observe their ECs, another measure of the geometry of the molecules. Whereas for wee1 there is on average less than one embranchment in the actives (EC_actives_ = 0.51), igf1r has more than two (EC_actives_ = 2.22) because its cavity, although similar, is less narrow than that of wee1, due to the presence of a methionine in its floor rather than a phenylalanine in wee1. Therefore, the fact that the decoys for both proteins have their average EC_decoys_ around 2.60 favors a good discrimination between actives and decoys for wee1 but not for igf1r. Again, in this case, the good performance for a target (wee1) may be imputable to the decoys.

For the last three targets, the analysis is more complex, because they did not obtain excellent BEDROC scores with all programs, as it could be expected. Glcm and reni stand out from the others for the same properties, HBD and RB, and they share high ∆EC (reni has additionally high ∆MW). They therefore obtained similar BEDROC scores, moderate with Glide and poor with FlexX. However, Surflex and Gold performed excellently for glcm (with scores higher than 0.88), and only moderately for reni (with scores around 0.45). To find an explanation, since they have similar actives/decoys biases, let us observe their cavities. For both proteins the cavities are moderately hydrophilic (1-FCA = 0.28 for glcm and 0.30 for reni) with more hydrogen-bond acceptors than hydrogen-bond donors, whereas the distribution of the hydrogen-bond acceptors are different: for glcm they are mostly in the depth of the cavity and for reni they are rather at the rim. In addition, for reni the cavity is more exposed to the solvent than for glcm (Exposure = 0.58 and 0.42, respectively). Therefore, for glcm, the active molecules being small (MW = 381 g/mol) and full of HBDs (on average 5.3), their relative high number of RBs (11.0) does not seem to penalize their placement and scoring with Gold and Surflex. However, for reni, the actives are the biggest in the DUD-E (MW = 558 g/mol), with the highest number of RBs (14.7), which makes their placement difficult. Their high number of HBDs (on average 4.3) is not sufficient to compensate this difficulty considering the distribution of the HB acceptors in the cavity. Hence the difference of performance for both proteins with Gold and Surflex.

The cavity of xiap is very exposed to the solvent (Exposure = 0.65). However, in this cavity, there is a small-volume hollow where the ligand anchors to the protein. Therefore, the active molecule should have several embranchments to be able to reach this hollow, while interacting with the rest of the cavity surface. Since the decoys lack some ramifications, they have more difficulty to bind stably to the protein. Hence the good BEDROC score with Glide and the medium ones with the other programs.

From what precedes in this section we conclude that the difference between decoys and actives does not guarantee the good performance of Gold, FlexX and Surflex. This good performance may take place only if the difference between the small molecule properties are complementary to the cavity characteristics.

#### …but necessary to obtain good performance from Gold, Surflex and FlexX

**Fig. 7:**
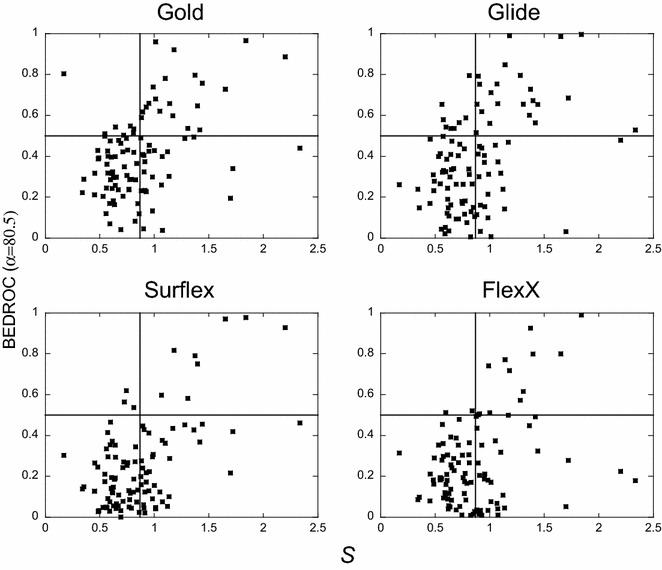
Programs performance with respect to the small molecule biases. For each target, the BEDROC score (α = 80.5) is plotted with respect to the sum, *S*, of the absolute values of the normalized differences between the actives and the decoys of all nine properties (see “Methods” section). The *vertical line* corresponds to the average of *S* over the 102 targets, 〈*S*〉 = 0.87, and the *horizontal line*, to BEDROC score = 0.5. In* each plot*, the* dots* in the *upper left* quadrant correspond to targets with low biases and high BEDROC scores

As observed from the stand-out targets, the important difference between the actives and decoys properties does not seem sufficient for the programs good performance, however, it may be necessary. To investigate it, for each target, the absolute values of the normalized differences of all nine properties were added (see “Methods” section) in order to discriminate between the targets for which there is no actives/decoys biases and the others. The average value over the 102 targets of these sums (*S*) is 0.87 and the median 0.81. All targets with *S* below the average were considered without bias. In the plots of Fig. 7 are reported the BEDROC scores of each program with respect to *S*. The horizontal line (at 0.5) delimits the good and poor scores and the vertical line (0.87) delimits the targets for which *S* is greater or smaller than the average value. In these plots, the presence of numerous points in the lower right quadrant (high bias, low performance) confirms the assertion concerning the fact that the existence of important biases in the chemical libraries is not sufficient to produce good scores. On the contrary, the almost total absence of points in the upper left quadrant (except for Glide) highlights the necessity of the presence of at least one important bias to yield good scores. Therefore, the chemical library biases seem insufficient but necessary to obtain good performances with Gold, Surflex and FlexX.

### Relationship between the program performance and the similarities between the actives and the crystal structure ligand

Because of the nature of the correlations, the previous section turned out to focus on decoys. In this section we will focus on the actives of each target, and more precisely on the similarities between them on the one hand and between them and the ligand in the crystal structure that was used for docking, on the other hand. For this purpose, the 2D fingerprints of the actives were generated using canvasFPGen module [30] from the Schrödinger suite, then the similarities between the small molecules were calculated using the Tanimoto method [32] and finally, for each target, the similarity values were averaged over all the actives. This was done for both the active–active comparison (Sim2D_act-act_) and the crystal-active comparison (Sim2D_cryst-act_). In both cases, the averages, Sim2D_act-act_ and Sim2D_cryst-act_, ranged between 0.0 and 0.4 (Additional file 1: Table S4). These similarities may seem small, but they can still be considered as significant because they correspond to averages over all actives. In addition, Sim2D between two molecules of the same family (with the same large core) may happen to be about 0.1, as it is the case for example of some actives of protein wee1.

The four targets that have the highest similarities between their actives, Sim2D_act-act_, are, in the descending order, wee1, sahh, glcm and pur2 and the four targets with the highest Sim2D_cryst-act_ are almost the same, wee1, kith, sahh and pur2. The three targets in common to these two small subsets, pur2, sahh and wee1, obtained very high BEDROC scores (over 0.72) with the four programs. Whereas pur2 and wee1 were discussed in the previous section because of the bias in their chemical libraries, sahh presented no important difference between the decoys and actives properties (see Additional file 1: Table S3). Its only important bias is in Sim2D_act-act_ and Sim2D_cryst-act_, showing that these similarities may play a significant role in the success of virtual screening.

The correlations between the program performance and both Sim2D_act-act_ and Sim2D_cryst-act_ were calculated (Fig. 5, lower panel). They showed that the performances of Glide, Gold and Surflex are dependent on the similarities between the actives, and those of Glide and Surflex on the similarities between these actives and the crystal structure ligand. The former correlation (with Sim2D_act-act_) is likely due to the same reasons as exposed above concerning the difference between the properties of actives and decoys. Indeed, it happened accidentally that when there is an important similarity between the actives, there is also an important number of hydrogen bond donors in these actives; the correlation between Sim2D_act-act_ and HBD_actives_ is 0.36 with *p*-value = 10^−4^. However, there is no significant correlation between Sim2D_cryst-act_ and HBD_actives_ or any other property. Therefore, in what follows, we will focus on Sim2D_cryst-act_.

Since all the virtual screenings performed here were done with flexible ligands but rigid protein targets, it is not surprising that the best successes (as for pur2, sahh and wee1) were obtained for targets whose actives are similar to the ligand of the crystal structure. Indeed, this similarity facilitates the positioning of the ligands, knowing that the protein structure is already in a conformation adapted to bind this type of molecules, which meets the observations made upon cross-docking actives in several crystal structures of the same protein [33]. Although this remark could concern all four programs, we observed discrepancies in their performances with respect to Sim2D_cryst-act_. Indeed, whereas the BEDROC scores of Glide and Surflex were correlated to Sim2D_cryst-act_, with high scores for all the targets with high Sim2D_cryst-act_ values (Glide high scores were obtained for Sim2D_cryst-act_ > 0.17 and Surflex’s for Sim2D_cryst-act_ > 0.24), this was not the case of Gold and FlexX, where all targets with high Sim2D_cryst-act_ did not obtain high scores. What is common between Gold and FlexX is that their positioning of the ligand is rather free, using a genetic algorithm for Gold and based on ligand fragments for FlexX, while it is made in a certain framework for Glide and Surflex, i.e., within a grid for Glide and using a protomol that fits the cavity in Surflex. Therefore, the relationship between the program performance and the similarities with the crystal structure ligand may be imputable to the positioning procedure of the program.

### What remains when all biases are removed from the benchmarking analysis

Let us only consider the targets for which there are no observed biases, i.e., without any small molecule property which would artificially favor the good performance of the programs. These targets have the sum of the differences of their small molecule properties *S* < 0.87 and Sim2D_cryst-act_ < 0.1; their number is 47. Of these targets, Glide performance was good (score > 0.5) for only 5 (in the descending score order: rxra, parp1, kif11, pa2ga and plk1), Gold, for 4 targets (fgfr1, kif11, rxra and fak1), Surflex, for 2 targets (ada17 and rxra) and FlexX for 2 targets (vgfr2, abl1). Therefore, considering Sim2D_cryst-act_ as a bias contributes to diminish still more the number of targets in the upper left quadrants of Fig. 7 and therefore, the percentage of targets without bias. In the beginning of this study, for α = 80.5, and considering the 102 DUD-E targets, we observed that Glide succeeded for 29 % of the targets (Additional file 1: Figure S1), Gold for 26 %, FlexX for 14 % and Surflex for 11 %. But if we rule out all biases and only consider the 47 targets, these proportions drop dramatically: Glide succeeds for only 11 % of the targets, Gold for 9 % and FlexX and Surflex for 4 %.

## Conclusion

From this study we may retain that, whatever the program, Gold, Glide, Surflex and FlexX, the VS performance is rather medium, since BEDROC scores >0.5 were obtained for less than half the proteins of the DUD-E data set. There are programs that seemed to perform better than others, like Gold and Glide. However, it appears that the good performance of a program in discriminating between active molecules and decoys is mainly due to biases, although small, either in the construction of the chemical libraries or in the similarities between the actives and the crystal structure ligand. Therefore, the meaning of program performance classification has to be questioned. However, based on this VS benchmark, we propose some properties of the programs that could be of interest. Glide, and to a lesser extent Surflex, appear to be the most efficient programs to retrieve ligands close to the crystal structure, due to their positioning algorithms. This will be shown in more details in an upcoming article. Concerning their scoring functions, Glide had no special sensitivity to any cavity or small molecule property, whereas the three other programs were mainly sensitive to the difference in hydrogen bond donors between decoys and actives. FlexX showed sensitiveness for electrostatic properties, whether they be for the cavities or for the real actives. This may indicate that in its scoring function, there is a bigger weight for the ionic interaction compared to the other three programs.

As to the idea that the benchmark of VS programs may help for creating a strategy to be used for certain protein families, it seems to us rather unrealistic. Indeed, in this work, with all four programs, only one family obtained good results, the protease family, and we have shown that the quality of these results were unfortunately mainly due to biases in the chemical libraries. Therefore, when a VS must be done for a given target, it would be better to use several different programs and combine their results rather than rely on the results of only one program. For example, our vSDC approach, which used the knowledge from these aforementioned programs, has proven to be successful in retrieving actives in an early recognition problem [24].

## Methods

### Preparation of the DUD-E database

This study was based on the 102 protein targets of the DUD-E [17] dataset, which is available on the web server http://dude.docking.orghttp://dude.docking.org/. It includes, for each target, a pre-processed PDB file and a set of active compounds and decoys, with an average actives/decoys proportion of 2 %.

#### Preparation of the DUD-E targets and definition of the binding sites

On the DUD-E website, it was described that some of the PDB files were automatically prepared while others were manually inspected by the DUD-E team. However, we observed some errors in the structures, as for instance the presence of hydrogens in disulfide bridges. We therefore processed all targets using the “Protein preparation wizard” from the Schrödinger suite (http://www.schrodinger.com) combined to the visual inspection of each target. Based on this preparation, the side chain orientation of glutamine, asparagine and histidine residues and the protonation state of histidine were determined. All hydrogen atoms were generated and the net charges of metal ions were assigned. The crystal water molecules that were considered as important by the DUD-E team, were conserved in the cavities, but cofactors that were not in the binding site were removed and only cofactors that were part of a cavity were included in the final structure. Finally, for each target, a mol2 file with suitable atom types was exported and used in the four docking programs.

To define the binding site, all residues with at least one heavy atom within 5 Å from the ligand of the target crystal structure were selected. Then upon individual visual inspection, when necessary, the selection was refined by adding every residue beyond 5 Å that was essential for the continuity of the cavity.

#### Preparation of the active compounds and decoys

Since the DUD-E database includes the set of actives and decoys in multi-mol2 format with the correct protonation state and 3D representation, we used them without any modification.

### Docking methods

For this benchmark, in order to obtain comparable results with all four programs, we used the VS method that they have in common, i.e., the rigid docking. In this docking the target atoms remain fixed whereas the small molecules are completely flexible. In order to prevent biases due to the setup of virtual screening calculations, we used the default parameters for all programs. The same procedure was followed for all targets.

#### Gold

Gold [19, 34] version 5.1 from the Cambridge Crystallographic Data Center (CCDC) was employed. The binding site residues were explicitly specified, as well as the metal ions coordination geometry. The latter was obtained by the prediction module of Gold, combined to the bibliographic information about the ion and its surrounding amino acids.

The positioning of small molecules in the binding site was based on the genetic algorithm, whose parameters were set to auto mode, using the PLP scoring function. The molecule ranking was based independently on either Goldscore or ChemPLP scoring functions. Goldscore function was preferred to ChemPLP despite the Gold user guide recommendations, because the average DE_2 %_ obtained from the VS with ChemPLP was equal to 16.37 % against 24.93 % for Goldscore. For this reason, the only results reported here are based on the Goldscore function.

#### Glide

Glide [20, 35] version 6.3 (Schrödinger) was used. Glide docking requires the generation of a cuboid grid centered on the binding site. For this purpose, the rotation of the target was done when necessary and the grid dimensions were adjusted visually to fit the cavity shape. The standard precision (SP) docking mode, with the default parameters, and the Glidescore scoring function, both recommended for virtual screening, were used.

#### Surflex

VS was performed with the Surflex [21] version 2.745 released by BioPharmics, LLC. The docking procedure necessitates the generation of a “protomol”, which consists of a set of hydrophobic and hydrophilic probes (CH4, NH and CO) that completely fit the cavity surface, making all possible interactions with the binding site residues. The generation of the protomol was based on the residue list previously defined, in addition to two parameters, the threshold and the “bloat”, which determine its stretch. The threshold was set to the default value (0.5), and the bloat to 2, which is in the range of the recommended values. For all targets, the resulting protomols, which are used to position the small molecules, were large enough to cover the entire cavity surface. The docking mode GeomX and the soft grid treatment mode (no scoring penalty for the ligand outside the box) were employed and the Surflex scoring function was used for the ranking of the molecules.

#### FlexX

FlexX [22] version 2.1.5 (BiosolveIT) was used. The binding site residues were explicitly specified, as well as the metal ions coordination geometry, similarly to what was done for Gold. In FlexX, the small molecule positioning algorithm is fragment based. The selection of the base fragments was set to automatic mode by using the “selbas a” option, where “a” stands for “automatic”, and the placement of the fragments used the standard algorithm (option 3). Each resulting pose was optimized by up to 1,000 steps energy minimizations with an additional cutoff distance of 3 Å to allow more interaction partners. The molecule ranking was done using the FlexX scoring function.

### Docking enrichment, net balance and BEDROC scores

The docking enrichment, DE, and the net balance, Δ*p*(a/b), were calculated using home-made scripts, whereas the BEDROC scores were calculated using the package Enrichvs proposed by Yabuuchi et al. [36], written in the R language [37].

### Target characteristics

#### Target families

In order to cluster the targets into families, we used the PDBAA database available on the NCBI web server, http://www.ncbi.nlm.nih.gov/books/NBK62345/. This database contains the protein sequence of all the PDB entries. The sequences of the 102 targets of the DUD-E were extracted from this database and aligned using the program Clustal Omega [25] available on the web server of the EBI (European Bioinformatics Institute, http://www.ebi.ac.uk/Tools/msa/clustalo/). The target pairs with more than 20 % sequence identity were visualized and homologous structures with similar functions were gathered in families.

#### The binding site properties

The hydrophobicity of the cavity was calculated by measuring the fraction of carbons among heavy atoms at the surface of the binding site. In order to define the surface, we used the protomol generated by Surflex as described above, since it closely fits the binding site and may therefore be representative of its surface. All heavy atoms of the protein situated at less than 4 Å from the protomol, i.e., in close contact with it, were considered as the surface of the cavity.

The SiteMap package [27] version 3.3 (Schrödinger) was used to calculate the exposure of the binding site, which measures the degree of openness of the site to solvent.

The size of the binding site was also calculated using SiteMap, but this property was not retained. Instead we considered the total number of heavy atoms that are located at 4 Å from the protomol, as described above.

### Small molecule characteristics

#### Molecule descriptors calculation

365 physicochemical properties for each small molecule were calculated using canvasMolDescriptors [30] module from the Schrödinger suite. Eight of the nine properties, MW, AlogP, E-state, PSA, HBA, HBD, RC and RB, were directly obtained from the program, whereas EC was calculated from some other descriptors given by the program. Indeed, the embranchment count was calculated by adding, for each molecule, the number of atoms involved in 3 or more covalent bonds, corresponding to the Canvas descriptors: ssssC_Cnt, sssCH_Cnt, sssNH_Cnt, sssN_Cnt, ssssN_Cnt, dssSe_Cnt, ssssssSe_Cnt, ddssSe_Cnt, sssSnH_Cnt, ssssSn_Cnt. As can be observed from this list, not only the organic atoms, C and N, were taken into account, but also more exotic ones, like Se and Sn. EC may be compared to the Chiral Center Count, which is directly given by canvasMolDescriptors. Both descriptors may be representative of embranchments at some extent, and they highly correlate (with *r* = 0.97), but we preferred to only keep EC, because it is based on geometrical considerations, close to graph theory.

For each of the nine retained properties, *P*, a normalized difference between actives and decoys was calculated as follows:

$$ \Delta P = \frac{{P_{actives} - P_{decoys} }}{{P_{actives}^{Max} - P_{actives}^{{min} } }} $$

and the sum of the absolute values of all 9 normalized property differences, Δ*P*_*i*_, was calculated:

$$ S = \sum\limits_{i = 1}^{9} {\left| {\Delta P_{i} } \right|} $$

The mean and median values of *S* over the 102 targets are 0.87 and 0.81, respectively.

#### 2D similarities

To calculate Sim2D_act-act_ and Sim2D_cryst-act_, first the 2D fingerprints of the molecules were calculated using the canvasFPGen [30] module, then the similarities were estimated using the Tanimoto method [32], all from the Schrödinger suite. For canvasFPGen, the default settings were conserved, i.e., the linear fingerprint type with a 32 bits precision. Considering the Tanimoto method, the similarities range between 0 (no similarity) and 1 (same molecule). The similarities were then averaged, for each target, over the entire set of actives.

#### Correlations

The Pearson correlation coefficients with the Student *t* test were calculated using the R program [37].

## Additional file

10.1186/s13321-016-0167-x All Tables and Figures of the Additional file.

## Abbreviations

VS: virtual screening
DUD-E: database of useful decoys-enhanced
DE: docking enrichment
BEDROC: Boltzmann enhanced discrimination of receiver operating characteristic
FCA: fraction of carbon atoms
MW: molecular weight
E-state: electrotopological state
PSA: polar surface area
HBA: hydrogen bond acceptors
HBD: hydrogen bond donors
RC: ring count
RB: rotatable bonds
EC: embranchment count
