# Supplementary material for: Benchmark of four popular virtual screening programs: construction of the active/decoy dataset remains a major determinant of measured performance
Source: J Cheminform. 2016 Oct 17;8:56. doi: 10.1186/s13321-016-0167-x (PMC5066283; doi:10.1186/s13321-016-0167-x)
Supplement: Supplementary file 1 — 10.1186/s13321-016-0167-x All Tables and Figures of the Additional file. [file 13321_2016_167_MOESM1_ESM.pdf]

Table S1. Bedroc scores ($\alpha = 80.5$) obtained with Gold, Glide, Surflex and FlexX and colored by family (see Table S2).

Target	Family	Gold	Glide	Surflex	FlexX
aa2ar	GPCR	0.29	0.13	0.34	0.17
abl1	Protein kinases	0.48	0.32	0.35	0.51
ace	Miscellaneous	0.30	0.10	0.09	0.03
aces	Miscellaneous	0.37	0.16	0.12	0.03
ada	Miscellaneous	0.32	0.12	0.19	0.18
ada17	Miscellaneous	0.35	0.46	0.56	0.05
adrb1	GPCR	0.43	0.31	0.25	0.18
adrb2	GPCR	0.43	0.50	0.41	0.36
akt1	Protein kinases	0.42	0.24	0.05	0.11
akt2	Protein kinases	0.62	0.41	0.24	0.27
aldr	Miscellaneous	0.40	0.39	0.14	0.21
ampc	Miscellaneous	0.04	0.09	0.00	0.04
andr	Nuclear receptors	0.04	0.37	0.08	0.01
aofb	Miscellaneous	0.29	0.15	0.15	0.10
bace1	Cleaving enzymes	0.43	0.26	0.27	0.19
braf	Protein kinases	0.43	0.56	0.15	0.51
cah2	Miscellaneous	0.29	0.08	0.04	0.17
casp3	Miscellaneous	0.53	0.56	0.37	0.49
cdk2	Protein kinases	0.30	0.47	0.07	0.24
comt	Miscellaneous	0.68	0.71	0.05	0.17
cp2c9	CYP450	0.12	0.04	0.05	0.19
cp3a4	CYP450	0.21	0.17	0.13	0.08
csf1r	Protein kinases	0.36	0.32	0.05	0.45
cxcr4	GPCR	0.08	0.01	0.27	0.01
def	Miscellaneous	0.49	0.18	0.62	0.01
dhi1	Miscellaneous	0.17	0.15	0.13	0.02
dpp4	Miscellaneous	0.29	0.18	0.09	0.21
drd3	GPCR	0.18	0.04	0.15	0.06
dyr	Miscellaneous	0.66	0.40	0.41	0.20
egfr	Protein kinases	0.42	0.40	0.24	0.35
esr1	Nuclear receptors	0.41	0.79	0.45	0.50
esr2	Nuclear receptors	0.32	0.75	0.43	0.51
fa10	Proteases	0.74	0.31	0.31	0.74
fa7	Proteases	0.80	0.73	0.79	0.92
fabp4	Miscellaneous	0.62	0.45	0.12	0.37
fak1	Protein kinases	0.51	0.34	0.12	0.31
fgfr1	Protein kinases	0.80	0.26	0.30	0.31
fkb1a	Miscellaneous	0.23	0.62	0.22	0.01
fnta	Miscellaneous	0.30	0.11	0.05	0.12
fpps	Miscellaneous	0.96	0.01	0.04	0.08
gcr	Nuclear receptors	0.13	0.21	0.30	0.18
glcm	Miscellaneous	0.89	0.48	0.93	0.22
gria2	Ion channels	0.24	0.44	0.13	0.12
grik1	Ion channels	0.42	0.31	0.19	0.36
hdac2	Histone deacetylases	0.31	0.17	0.25	0.30
hdac8	Histone deacetylases	0.20	0.09	0.08	0.27
hivint	Miscellaneous	0.19	0.03	0.22	0.05
hivpr	Miscellaneous	0.30	0.14	0.10	0.05
hivrt	Miscellaneous	0.42	0.37	0.13	0.19
hmdh	Miscellaneous	0.40	0.66	0.38	0.04
hs90a	Miscellaneous	0.23	0.03	0.02	0.03
hxk4	Miscellaneous	0.30	0.34	0.04	0.08

Table S1. continued

Target	Family	Gold	Glide	Surflex	FlexX
igf1r	Protein kinases	0.49	0.52	0.20	0.49
inha	Miscellaneous	0.51	0.27	0.21	0.18
ital	Miscellaneous	0.12	0.10	0.02	0.03
jak2	Protein kinases	0.50	0.41	0.17	0.19
kif11	Miscellaneous	0.55	0.59	0.12	0.08
kit	Protein kinases	0.24	0.12	0.05	0.17
kith	Miscellaneous	0.26	0.75	0.60	0.07
kpcb	Protein kinases	0.49	0.60	0.43	0.45
lck	Protein kinases	0.26	0.41	0.19	0.39
lkha4	Miscellaneous	0.29	0.54	0.47	0.16
mapk2	Protein kinases	0.40	0.45	0.04	0.20
mcr	Nuclear receptors	0.04	0.30	0.06	0.01
met	Protein kinases	0.64	0.44	0.17	0.35
mk01	Protein kinases	0.46	0.26	0.03	0.39
mk10	Protein kinases	0.39	0.28	0.03	0.29
mk14	Protein kinases	0.24	0.34	0.06	0.29
mmp13	Miscellaneous	0.40	0.33	0.30	0.09
mp2k1	Protein kinases	0.39	0.23	0.03	0.16
nos1	Miscellaneous	0.40	0.16	0.35	0.14
nram	Miscellaneous	0.34	0.69	0.42	0.28
pa2ga	Miscellaneous	0.32	0.58	0.09	0.30
parp1	Miscellaneous	0.24	0.65	0.34	0.28
pde5a	Miscellaneous	0.16	0.30	0.19	0.05
pgh1	Cyclooxygenases	0.22	0.24	0.14	0.09
pgh2	Cyclooxygenases	0.32	0.48	0.27	0.21
plk1	Protein kinases	0.48	0.53	0.11	0.23
pnph	Miscellaneous	0.45	0.15	0.22	0.21
ppara	Nuclear receptors	0.32	0.19	0.37	0.09
ppard	Nuclear receptors	0.23	0.11	0.16	0.04
pparg	Nuclear receptors	0.32	0.15	0.12	0.04
prgr	Nuclear receptors	0.07	0.05	0.07	0.10
ptn1	Miscellaneous	0.78	0.32	0.36	0.32
pur2	Miscellaneous	0.97	1.00	0.98	0.99
pygm	Miscellaneous	0.17	0.02	0.03	0.12
pyrd	Miscellaneous	0.50	0.57	0.27	0.48
reni	Cleaving enzymes	0.44	0.53	0.46	0.18
rock1	Protein kinases	0.20	0.40	0.05	0.35
rxra	Nuclear receptors	0.53	0.79	0.54	0.27
sahh	Miscellaneous	0.73	0.99	0.97	0.80
src	Protein kinases	0.24	0.24	0.12	0.30
tgfr1	Protein kinases	0.59	0.66	0.28	0.44
thb	Nuclear receptors	0.54	0.54	0.29	0.26
thrb	Proteases	0.54	0.66	0.58	0.62
try1	Proteases	0.65	0.67	0.75	0.80
tryb1	Proteases	0.60	0.47	0.44	0.50
tysy	Miscellaneous	0.76	0.65	0.46	0.32
urok	Proteases	0.66	0.85	0.29	0.77
vgfr2	Protein kinases	0.36	0.26	0.07	0.52
wee1	Protein kinases	0.92	0.99	0.82	0.72
xiap	Miscellaneous	0.49	0.80	0.45	0.57

Table S2. Definition of the families. The color code is similar to Figures 3 and 4.

Family name (# of targets)	Target name	PDB code
1-Protein kinases (26)	abl1,akt1,akt2,braf,cdk2, csf1r,egfr,fak1,fgfr1,igf1r, jak2,kit,kpcb,lck,mapk2, met,mk01,mk10,mk14,mp2k1, plk1,rock1,src,tgfr1,vgfr2, wee1	2hzi,3cqw,3d0e,3d4q,1h00, 3krj,2rgp,3bz3,3c4f,2oj9, 3lpb,3g0e,2i0e,2of2,3m2w, 3lq8,2ojg,2zdt,2qd9,3eqh, 2owb,2etr,3el8,3hmm,2p2i, 3biz
2-Nuclear receptors (11)	andr,esr1,esr2,gcr,mcr, ppara,ppard,pparg,prgr,rxra, thb	2am9,1sj0,2fsz,3bqd,2aa2, 2p54,2znp,2gtk,3kba,1mv9, 1q4x
3-Proteases (6)	fa7,fa10,thrb,try1, tryb1,urok	1w7x,3kl6,1ype,2ayw, 2zec,1sqt
4-GPCR (5)	aa2ar,adrb1,adrb2,cxcr4,drd3	3eml,2vt4,3ny8,3odu,3pbl
5-Cleaving enzymes (2)	bace1,reni	3l5d,3g6z
6-Cyclooxygenases (2)	pgh1,pgh2	2oyu,3ln1
7-CYP450 (2)	cp2c9,cp3a4	1r9o,3nxu
8-Ion channels (2)	gria2,grik1	3kgc,1vso
9-Histone deacetylases (2)	hdac2,hdac8	3max,3f07
10-Miscellaneous (44)	aces,ace,ada17,ada,aldr, ampc,aofb,cah2,casp3,comt, def,dhi1,dpp4,dyr,fabp4, fkb1a,fnta,fpps,glcm,hivint, hivpr,hivrt,hmdh,hs90a,hxk4, inha,ital,kif11,kith,lkha4, mmp13,nos1,nram,pa2ga, parp1,pde5a,pnph,ptn1,pur2, pygm,pyrd,sahh,tysy,xiap	1e66,3bkl,2oi0,2e1w,2hv5, 1l2s,1s3b,1bcd,2cnk,3bwm, 1lru,3frj,2i78,3nxo,2nnq, 1j4h,3e37,1zw5,2v3f,3nf7, 1xl2,3lan,3ccw,1uyg,3f9m, 2h7l,2ica,3cjo,2b8t,3chp, 830c,1qw6,1b9v,1kvo, 3l3m,1udt,3bgs,2azr,1njs, 1c8k,1d3g,1li4,1syn,3hl5

Table S3. Summary of the most important properties for some particular targets. For the protease family the values were averaged over all the targets of this family. The quality of the BEDROC scores are shown under the target name, in the same order as in Table S1, i.e., **Gold, Glide, Surflex and FlexX**. ✓: good score (over 0.6), ×: moderate score (between 0.4 and 0.6) and –: poor score (under 0.4). The color code is as follows: dark green corresponds to the maximum value and dark orange to the minimum value. Light green in cavity properties: values higher than for the average of the targets. Light green/orange in small molecule properties and similarities: between the second and fifth highest/lowest values.

	Cavity properties		Small molecule properties				Similarities
	(1-FCA)	Expo	ΔPSA	ΔHBD	ΔEC	ΔRB	$Sim2D_{cryst\text{-}act}$
<Proteases><br>✓✓×✓	0.37	0.66	0.09	-0.20	-0.03	0.10	0.06
Pur2<br>✓✓✓✓	0.35	0.37	0.32	0.22	-0.04	0.20	0.27
Mk01<br>× – – –	0.35	0.37	-0.11	0.13	-0.17	0.00	0.17
Wee1<br>✓✓✓✓	0.31	0.43	-0.02	0.09	-0.34	-0.04	0.39
Igf1r<br>× × – ×	0.30	0.47	0.02	0.11	-0.09	-0.01	0.06
Glcm<br>✓× ✓–	0.28	0.42	0.08	0.56	0.49	0.36	0.01
Reni<br>× × × –	0.30	0.58	0.10	0.45	0.67	0.32	0.03
Xiap<br>×✓××	0.26	0.65	-0.03	0.06	0.63	0.07	0.11
Sahh<br>✓✓✓✓	0.35	0.33	0.08	0.14	0.36	-0.08	0.27

Table S4. Average of similarities, $Sim2D_{act-act}$ and $Sim2D_{cryst-act}$, for each target.

Target	$Sim2D_{act-act}$	$Sim2D_{cryst-act}$
aa2ar	0.08	0.02
abl1	0.07	0.06
ace	0.11	0.09
aces	0.07	0.03
ada	0.13	0.10
ada17	0.10	0.08
adrb1	0.08	0.09
adrb2	0.08	0.06
akt1	0.09	0.03
akt2	0.09	0.06
aldr	0.05	0.09
ampc	0.12	0.11
andr	0.04	0.05
aofb	0.06	0.04
bace1	0.07	0.04
braf	0.09	0.03
cah2	0.07	0.02
casp3	0.12	0.06
cdk2	0.05	0.05
comt	0.25	0.24
cp2c9	0.05	0.05
cp3a4	0.05	0.03
csf1r	0.08	0.09
cxcr4	0.22	0.07
def	0.12	0.10
dhi1	0.05	0.06
dpp4	0.06	0.04
drd3	0.07	0.04
dyr	0.07	0.06
egfr	0.10	0.11
esr1	0.06	0.12
esr2	0.06	0.07
fa10	0.07	0.06
fa7	0.12	0.13
fabp4	0.16	0.15
fak1	0.22	0.09
fgfr1	0.10	0.03
fkb1a	0.19	0.07
fnta	0.07	0.06
fpps	0.15	0.09
gcr	0.06	0.02
glcm	0.28	0.01
gria2	0.05	0.05
grik1	0.07	0.04
hdac2	0.08	0.10
hdac8	0.08	0.06
hivint	0.09	0.04
hivpr	0.08	0.05
hivrt	0.04	0.02
hmdh	0.11	0.10
hs90a	0.13	0.03
hxk4	0.13	0.08
igf1r	0.08	0.06
inha	0.21	0.14
ital	0.12	0.05
jak2	0.07	0.09
kif11	0.12	0.06
kit	0.07	0.06
kith	0.23	0.33
kpcb	0.12	0.08
lck	0.06	0.04
lkha4	0.13	0.13
mapk2	0.08	0.02
mcr	0.07	0.03
met	0.12	0.15
mk01	0.19	0.17
mk10	0.09	0.03
mk14	0.06	0.04
mmp13	0.09	0.11
mp2k1	0.13	0.11
nos1	0.07	0.02
nram	0.19	0.04
pa2ga	0.12	0.05
parp1	0.07	0.06
pde5a	0.06	0.08
pgh1	0.04	0.04
pgh2	0.05	0.07
plk1	0.12	0.03
pnph	0.10	0.11
ppara	0.11	0.08
ppard	0.12	0.12
pparg	0.09	0.08
prgr	0.05	0.05
ptn1	0.09	0.05
pur2	0.27	0.27
pygm	0.17	0.03
pyrd	0.11	0.18
reni	0.19	0.03
rock1	0.09	0.04
rxra	0.10	0.02
sahh	0.34	0.28
src	0.07	0.05
tgfr1	0.10	0.08
thb	0.16	0.11
thrb	0.07	0.03
try1	0.07	0.05
tryb1	0.13	0.04
tysy	0.12	0.07
urok	0.08	0.07
vgfr2	0.06	0.06
wee1	0.34	0.39
xiap	0.22	0.11

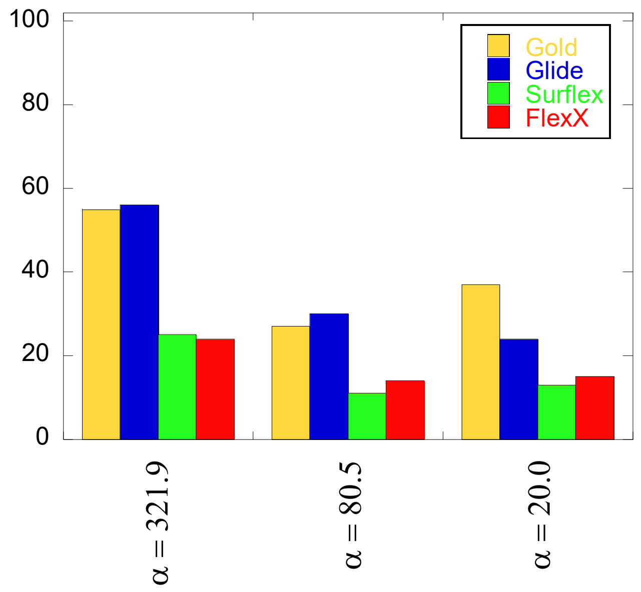

Figure S1. Number of targets with BEDROC scores > 0.5, with the three α parameters, for the four programs.

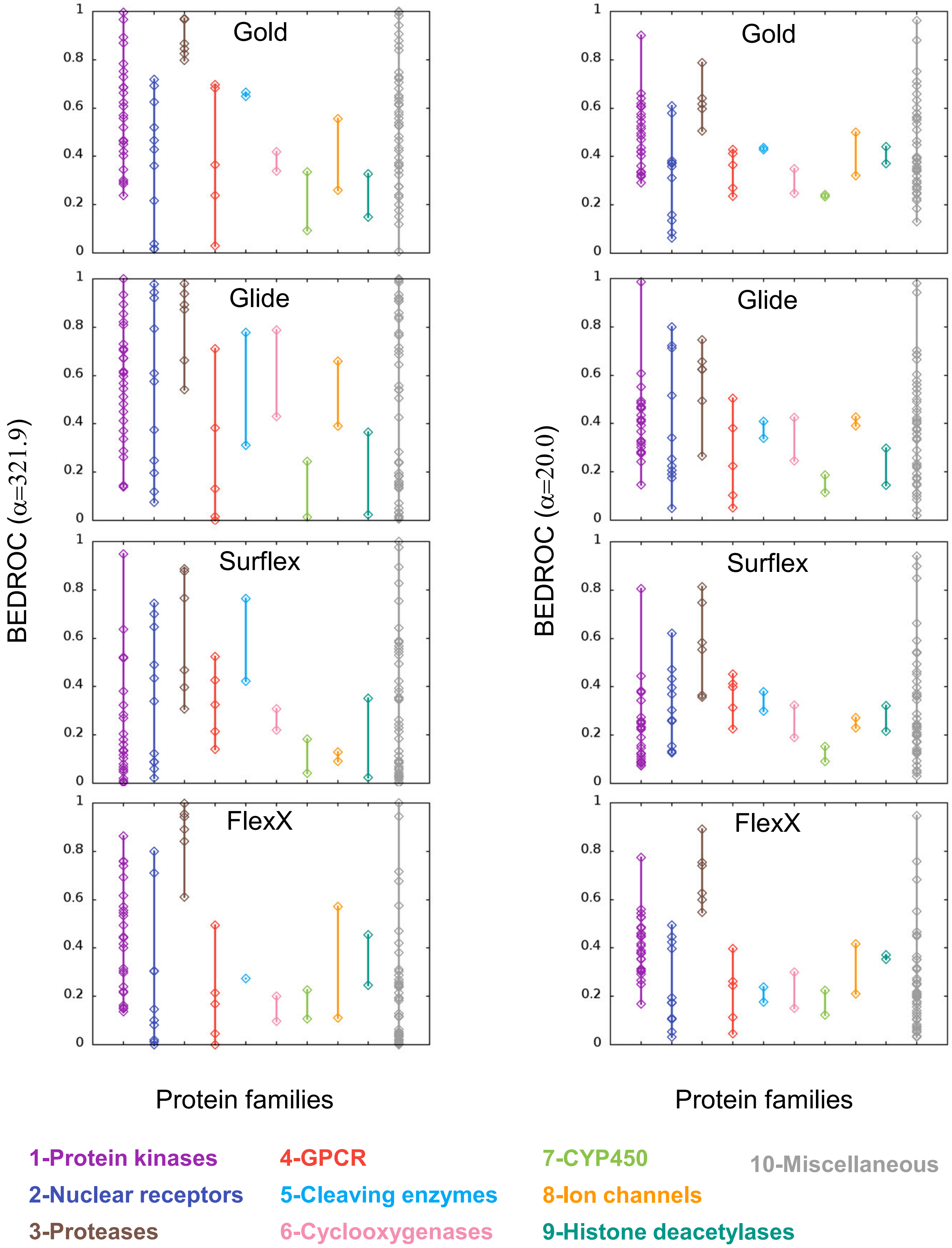

Figure S2. Same legend as Figure 3, for BEDROC scores with α=321.9 and α=20.0.

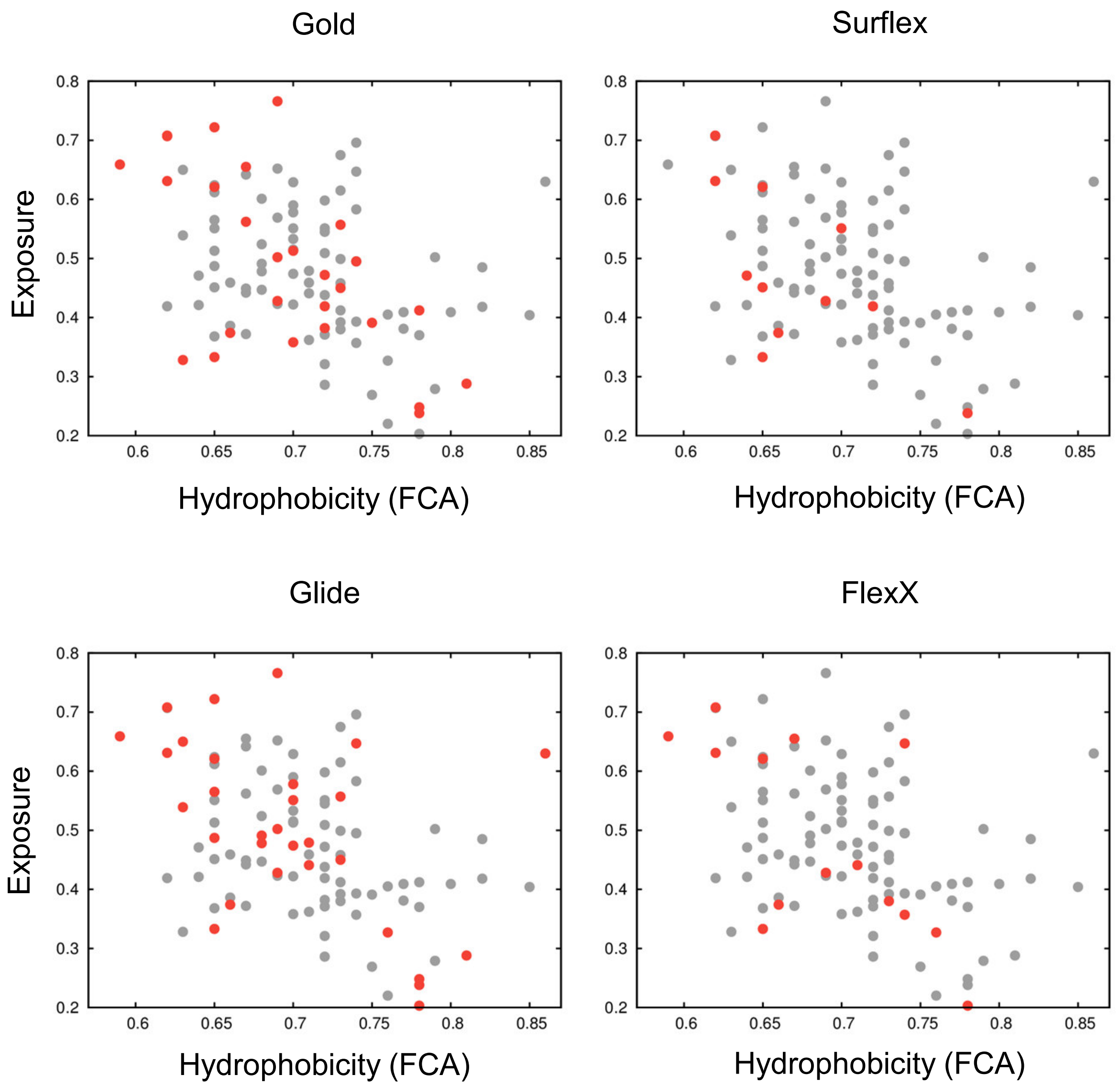

Figure S3. Distribution of the high BEDROC scores (α=80.5) with respect to the characteristics of the protein cavities. For each target, the exposure of the cavity is represented *vs* its hydrophobicity. The targest that obtained scores > 0.5 are colored in red.

α=321.9

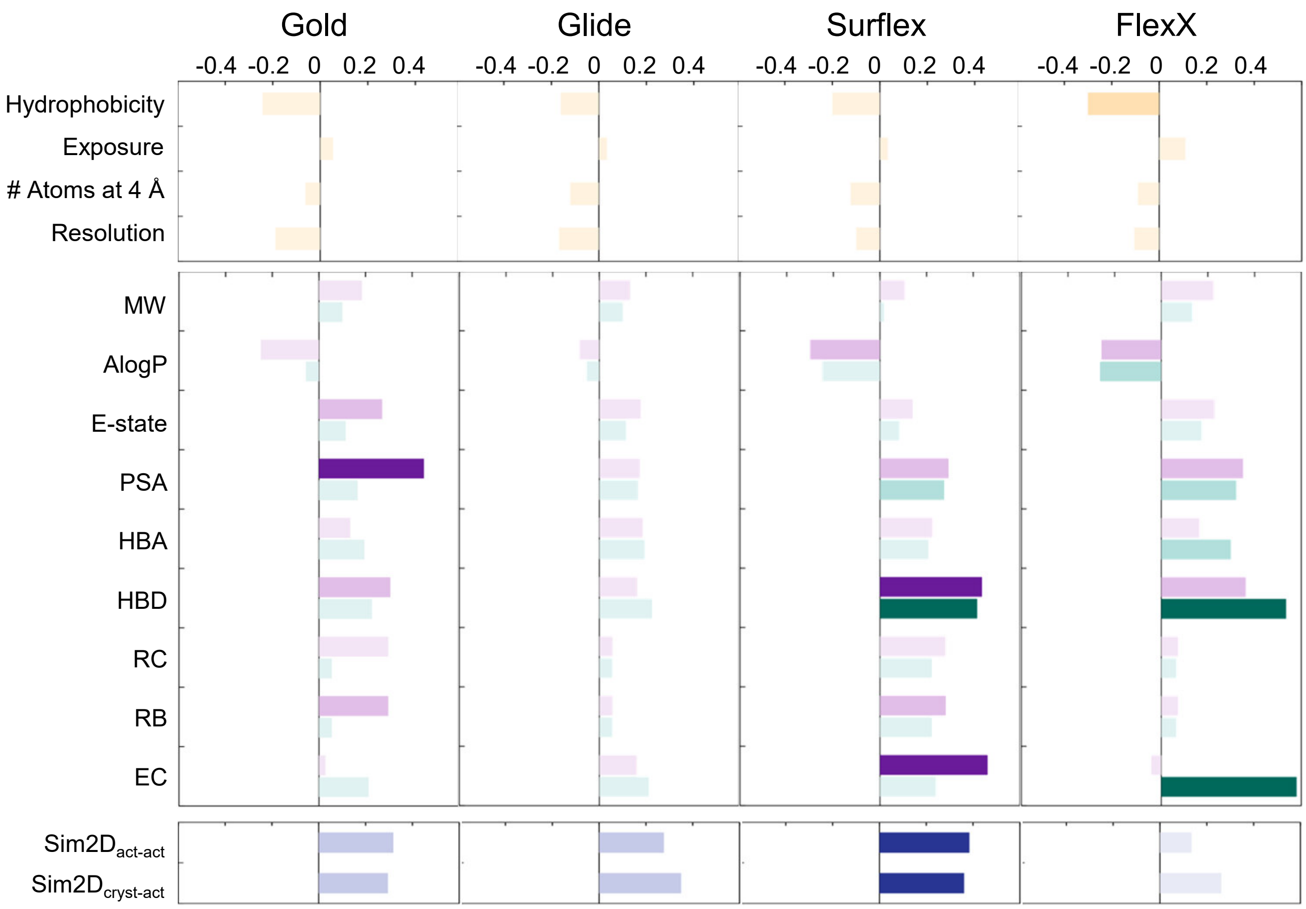

Figure S4. continued

α=20.0

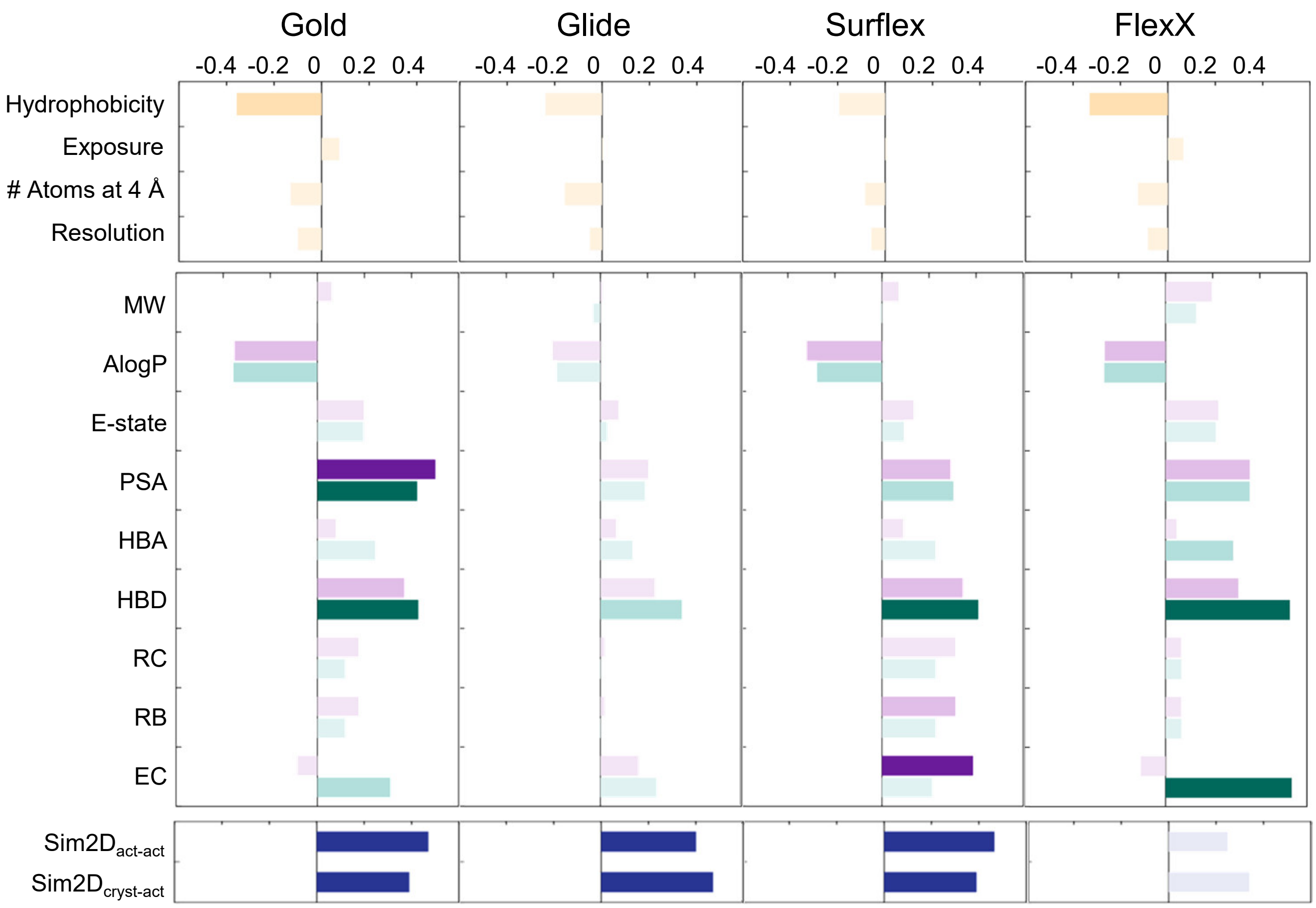

Figure S4. Same legend as Figure 5, for BEDROC scores with α=321.9 and α=20.0.
